# Quantitation of underivatized branched-chain amino acids in sport nutritional supplements by capillary electrophoresis with direct or indirect UV absorbance detection

**DOI:** 10.1371/journal.pone.0179892

**Published:** 2017-06-22

**Authors:** Jun Qiu, Jinhao Wang, Zhongqi Xu, Huiqing Liu, Jie Ren

**Affiliations:** 1 Shanghai Research Institute of Sports Science, Shanghai, China; 2 College of Chemistry, Chemical Engineering and Biotechnology, Donghua University, Shanghai, China; 3 China Table Tennis College, Shanghai University of Sport, Shanghai, China; Helsingin Yliopisto, FINLAND

## Abstract

The branched-chain amino acids (BCAAs) including leucine (Leu), isoleucine (Ile) and valine (Val) play a pivotal role in the human body. Herein, we developed capillary electrophoresis (CE) coupled with conventional UV detector to quantify underivatized BCAAs in two kinds of sport nutritional supplements. For direct UV detection at 195 nm, the BCAAs (Leu, two enantiomers of Ile and Val) were separated in a background electrolyte (BGE) consisting of 40.0 mmol/L sodium tetraborate, and 40.0 mmol/L β-cyclodextrin (β-CD) at pH 10.2. In addition, the indirect UV detection at 264 nm was achieved in a BGE of 2.0 mmol/L Na_2_HPO_4_, 10.0 mmol/L p-aminosalicylic acid (PAS) as UV absorbing probe, and 40.0 mmol/L β-CD at pH 12.2. The β-CD significantly benefited the isomeric separation of Leu, L- and D-Ile. The optimal conditions allowed the LODs (limit of detections) of direct and indirect UV absorption detection to be tens μmol/L level, which was comparable to the reported CE inline derivatization method. The RSDs (relative standard deviations) of migration time and peak area were less than 0.91% and 3.66% (*n* = 6). Finally, CE with indirect UV detection method was applied for the quantitation of BCAAs in two commercial sport nutritional supplements, and good recovery and precision were obtained. Such simple CE method without tedious derivatization process is feasible of quality control and efficacy evaluation of the supplemental proteins.

## Introduction

The branched-chain amino acids (BCAAs) including leucine (Leu), isoleucine (Ile) and valine (Val) have become a popular sports nutritional supplement for the athletes to meet their metabolic needs [1]. The BCAAs are type of essential amino acids (AAs) that could not be synthesized by human body itself, so sufficient BCAAs must be supplied in the diet or in the nutritional supplements [2]. Recent studies have demonstrated that BCAAs play an important role in the regulation of protein metabolism, *e*.*g*. to stimulate protein synthesis by activating mammalian target of repamycin (mTOR) and suppressing protein degradation by inhibiting autophagy [3]. In sports science, the benefits of intake of BCAAs on athletes are to prevent peripheral fatigue, delay central fatigue [4,5], and strengthen immune function [6]. Currently, the commercial market offers various sports nutrition supplements containing BCAAs, which have been widely used in the world. For the purpose of quality control and efficacy evaluation of sports nutrition supplements or nutraceuticals, developing new methods or improving current methods for the separation and quantification of BCAAs is still an area of concern.

So far, the chromatographic methods such as gas chromatography (GC) [7,8], high performance liquid chromatography (HPLC) [9–11] and capillary electrophoresis (CE) [12–15] have been mainly used for the separation of essential AAs or BCAAs in clinical or laboratory studies. The nonvolatile AAs are required to be derivatized prior to GC. Therefore, HPLC and CE seem to be more suitable analytical tools. Especially, CE is promising in many application areas due to technique’s primary advantages of high separation efficiency, simple instrumentation, minor consumption of sample and chemicals, and tolerance to complex matrices, *etc*. With respect to the detection strategies for BCAAs, MS (mass spectrometry) is the most powerful detector due to its high sensitivity, identification ability and mature connection with GC or HPLC [7–10]. CE-MS has also emerged as an effective tool for the analysis of twenty AAs with low LOD (limit of detection) at ng/mL level [16], but the disadvantage is ionization issue which due to CE-MS strongly depends on background electrolyte (BGE). Fluorescence detection (FD) is another sensitive analytical approach for the BCAAs, but precolumn derivatization is required since BCAAs are lack of intrinsic fluorescence. Chang *et al*. used CE-FD to quantify BCAAs in ascites with naphthalene-2,3-dicarboxaldehyde derivatization, and tens nmol/L of LOD were achieved [12]. The most universal detection approach is UV-Vis absorbance for the commercial CE apparatus. Thus, the UV-Vis detection of AAs seems more practical and simple in many applications. To address the issue of weak or none absorption of AAs in UV-Vis range, it is necessary to carry on offline or inline derivatization of AAs (including BCAAs) with different chemical reagents [13,14,17,18]. Unfortunately, the derivatization process of AAs must be carefully controlled, which can lead to the targets are detected not in their native states. Therefore, the separation and detection of underivatized AAs still face challenges. Contactless conductivity detector (CCD) is an optional method and the LOD range is from tens to sub μmol/L [15,19,20]. Few reports except Crespo et al. have described the glutamic acid, glycine and alanine without derivatization were detected by the direct CE-UV detection (at 220 nm) [21]. In addition, many studies have showed that indirect UV absorbance detection is an important and alternative tool for the analysis of AAs in their native state [22], but has not been successfully applied in the separation of BCAAs.

The present study described a simple and low cost CE method for the determination of underivatized BCAAs with direct or indirect UV detection. For this purpose, two optimal BGEs were respectively proposed to accommodate the isomeric separation of the targets. It was demonstrated the direct UV detection was not suitable for the real sample analysis due to the existing strong absorbance interference. Therefore, the indirect UV detection was for the first time to analyze not only the standard BCAAs but also the real supplemental proteins. The obtained results might be promising for the analysis of BCAAs in human bioliquids to direct the usage of the supplemental proteins, for the purpose of ensuring BCAAs needs but not exceeding tolerable upper intake level.

## Materials and methods

### CE system

CE analyses were performed on a P/ACE^TM^ MDQ system (Beckman Coulter, CA, USA) equipped with a photo-diode array (PDA) detector, with which the detection wavelength could be set from 195 to 600 nm. The data acquisition and treatment was conducted by 32 Karat Software. The temperature of the capillary cartridge was maintained at 25°C by an insert coolant liquid. Separations were carried out in a 60 cm long (effective length 50 cm) and 75 μm I.D. fused-silica capillary from Ruifeng Chromatographic Co., Ltd. (Hebei, China). Before use, the new capillary was preconditioned with 0.1 mol/L NaOH (5 min) and water (5 min). Prior to each run, the capillary was flushed with BGE (3 min). The sample was introduced by applying a pressure of 0.5 psi for 5 s. After sample injection, the separation was conducted by applying a constant voltage of +15.0 kV, and the PDA wavelength was setting at 195 nm and 264 nm for direct and indirect UV detection, respectively.

### Reagents and solutions

All chemicals were of analytical grade. The p-aminosalicylic acid (PAS), sodium tetraborate, Leu and Ile were supplied by Sigma-Aldrich (MO, USA). Val and L-Ile were from J&K Chemical (Beijing China). The used Na_2_HPO_4_, β-cyclodextrin (β-CD) and NaOH were provided by Sinopharm Group Co., Ltd. (Shanghai China). The stock solution of BCAAs was prepared at 10.0 mmol/L and stored in a refrigerator at 4.0°C. The optimized BGE for direct UV detection was 40.0 mmol/L sodium tetraborate and 40.0 mmol/L β-CD at pH 10.2 adjusted with 1.0 mol/L NaOH. For indirect UV detection, PAS was added in BGE as UV absorbing probe. The BGE was 2.0 mmol/L Na_2_HPO_4_, 10.0 mmol/L PAS, and 40.0 mmol/L β-CD at pH 12.2 adjusted with 1.0 mol/L NaOH. All solutions were prepared in purified water obtained by a Milli-Q Labsystem (Millipore, Germany), and filtered through a 0.45 μm membrane before electrophoretic run.

### Treatment of the supplemental proteins

Two commercial sports nutritional supplements with different flavors, vanilla and chocolate type, were from Shanghai Sports Science Institute (Shanghai, China). It was noticed the samples were made of high quality whey protein and used for athletes in order to improve their performance. For the analysis of the AAs composition in proteins, various methods for the hydrolysis of proteins have been evaluated [23]. Herein, we used acidic solution to hydrolyze the supplemental proteins. All samples with 1.0 g weight were accurately weighed and hydrolyzed by 6.0 mL HCl (6 mol/L) at 110°C for 24 h under the atmosphere of nitrogen. The obtained hydrolysate was neutralized by 1.0 mol/L NaOH, and then carried out the centrifugation at 10000 rpm for 10 minutes. The precipitation was washed and centrifuged for 3 times. All supernatants were collected in a 50.0 mL volumetric flask, diluted with purified water to the volume, and stored at -20°C. Prior to CE analysis, the hydrolysate was filtered by a 0.45 μm membrane.

## Results and discussion

### Optimization of direct UV detection

In general, AAs need to be derivatized when direct UV detection is adopted, except one previously report by Crespo et al. [21]. We checked the UV absorption spectrum of BCAAs, which owned a certain UV absorbance below 200 nm. It implied BCAAs might be detected by direct UV absorbance in a suitable BGE. In consideration of BGE absorbance at the low UV range, the sodium tetraborate buffer system was better than carbonate and phosphate. Afterwards, the concentration of sodium tetraborate and pH of BGE (adjusted by NaOH) were investigated. The concentration of sodium tetraborate was checked from 10 to 50 mmol/L. The results showed that high concentration of sodium tetraborate could improve the separation efficiency, but caused high current and serious Joule heat in capillary that resulted in deterioration of separation. So the concentration of sodium tetraborate was 40 mmol/L. The pH value of BGE affected electroosmotic flow (EOF) and the dissociation of BCAAs. In order to obtain fully negatively charged BCAAs and keep sufficient buffer capacity, the final pH value was determined at 10.2.

**Fig 1:**
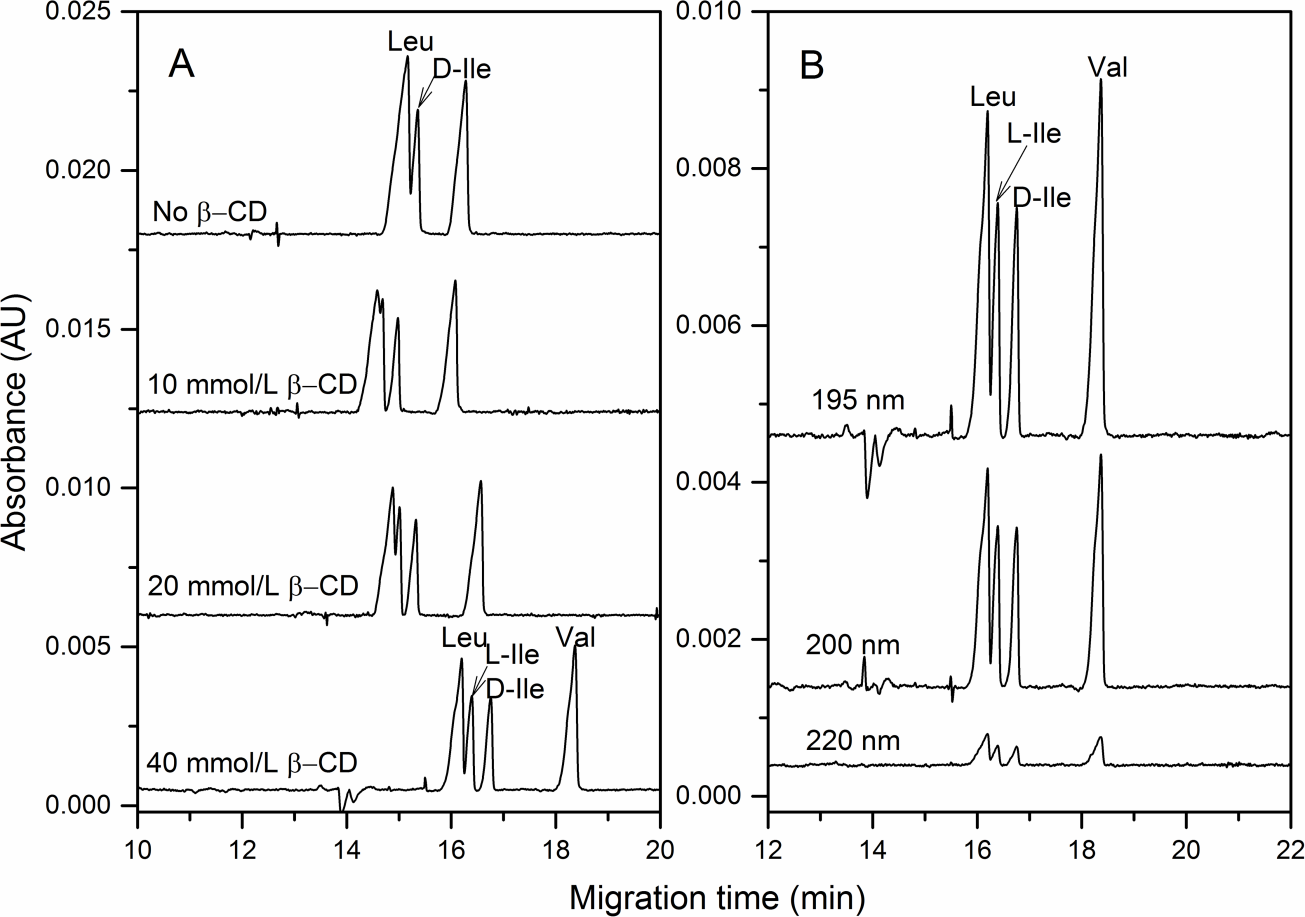
Separation of BCAAs affected by the concentration of β-CD (A) and the wavelength (B) at the direct UV detection mode. The optimization process in A was obtained by adding β-CD in 40.0 mM sodium tetraborate (pH 10.2), and detected at 195 nm. The separation profiles in B were at optimal BGE. Capillary: 60 cm long (effective length 50 cm) and 75 μm I.D. Sample: 2.0 mmol/L Leu, Ile and Val. Sample introduction: 0.5 psi for 5 s. Separation voltage: +15 kV.

In our targets, Leu and Ile were two regioisomers, and Ile contained a pair of enantiomers as D- and L-Ile. Some chiral selectors were trialed in order to achieve enantioseparation of Leu, D- and L-Ile. Here, the β-CD was conducted as a cheap and valid chiral selector, which was demonstrated in Fig 1A. The higher concentration of β-CD, the better enantioseparation was achieved. Since the issue of water solubility, 40 mmol/L β-CD can already achieve the best enantioseparation. If with 50 mmol/L β-CD, the separation was not improved. When using the optimal BGE, the separation efficiency of Leu and L-Ile were much improved but still not baseline separated (resolution around 0.8). Another important parameter for direct UV detection was wavelength. Three wavelengths (195, 200 and 220 nm) were selected to check the separation profiles as displayed in Fig 1B. Obviously, the peak response of BCAAs became stronger with the decreasing of wavelength. Therefore, the direct UV detection was carried out at 195 nm, which was the allowable minimum wavelength of the PDA detector.

In Table 1, the analytical performance characteristics as repeatability, linear range and LODs (at *S/N* = 3) were listed. Only L-Ile was quantified since Ile in the nutrition exclusively existed as L-Ile according to several studies [13,24]. For direct UV detection, the achieved LODs were averaged at 32.6 μmol/L. In comparison of inline derivatization method, o-phthalaldehyde (OPA) labled BCAAs were detected at 340 nm [17], and the obtained LODs here were close but no advantage. However, our process was simple and time saving. The RSDs (relative standard deviations) of migration time and area were less than 0.4% and 3.5% (n = 6), respectively. It is demonstrated that the direct UV detection is applicable for qualitative and quantitative analysis of BCAAs.

**Table 1:** Analytical parameters for direct and indirect UV detection of BCAAs.

UV detection mode	Analytes	Repeatability (%RSD, n = 6)		Regression equation (R^2^)^a^	Calibration range (μmol/L)	LOD^b^ (μmol/L)
		Migration time	Peak area			
Direct	Leu	0.4	1.7	y = 4.1595x-0.1632 (0.9917)	200~2000	37.2
	L-Ile	0.4	3.5	y = 3.2015x-0.3065 (0.9924)	200~2000	35.9
	Val	0.4	2.4	y = 4.0913x+0.3425 (0.9932)	200~2000	24.6
Indirect	Leu	0.8	2.4	y = 6.9669x+0.00178 (0.9995)	50~1000	11.8
	L-Ile	0.9	3.7	y = 7.5604x-0.13346 (0.9996)	50~1000	11.1
	Val	0.9	3.2	y = 12.5059x+0.16675 (0.9987)	50~1000	7.3

^a^ X represents concentration (mmol/L), and y represents peak area (AU•min×10^−4^).

^b^ LODs were calculated at a suitable concentration that the peak offered S/N value less than 10.

### Optimization of indirect UV detection

**Fig 2:**
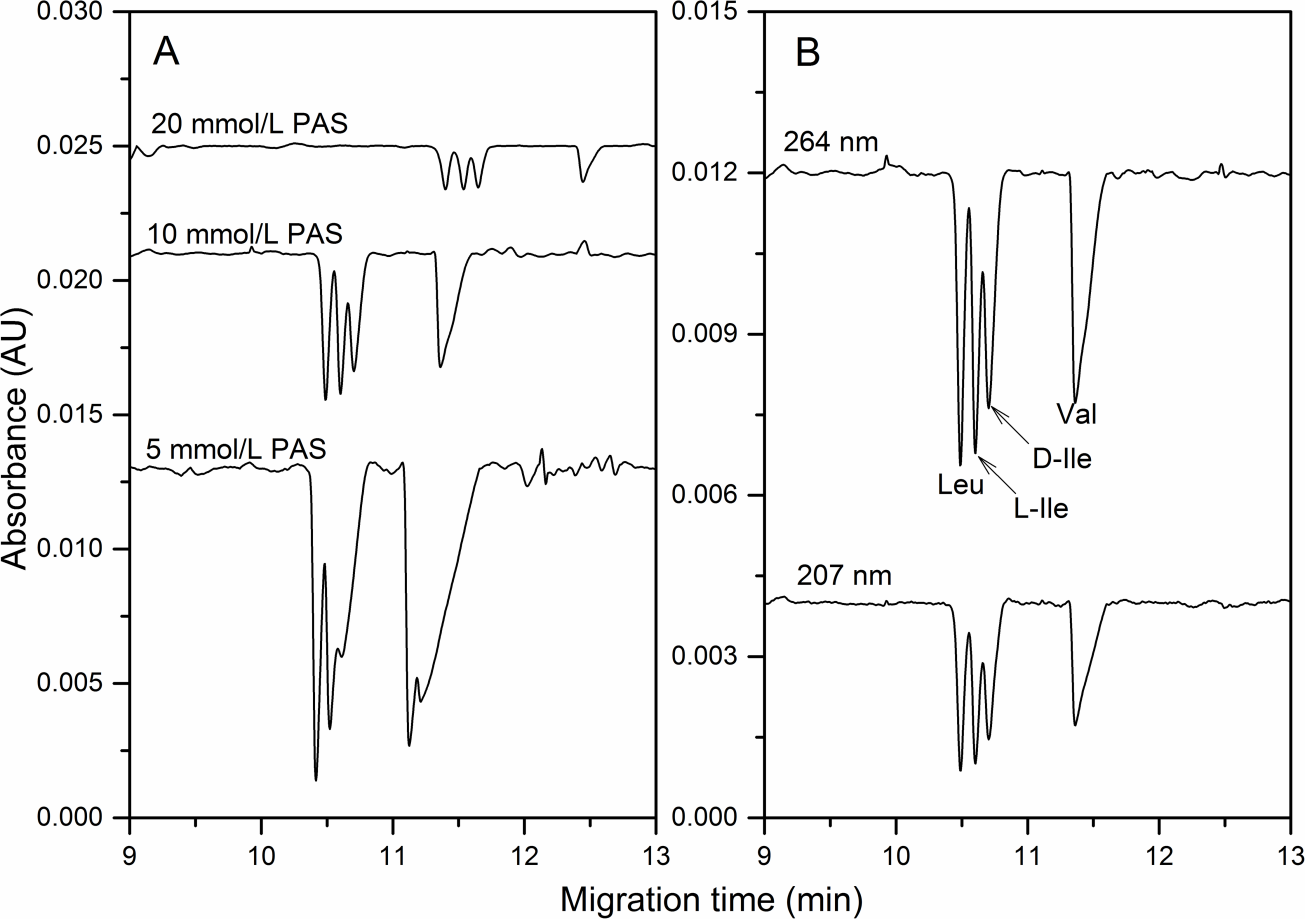
Indirect UV detection of BCAAs affected by the concentration of PAS (A) and the wavelength (B). The optimization process in A was obtained by different PAS concentration in 40.0 mM β-CD, 2.0 mmol/L Na_2_HPO_4_ (pH12.2), and detected at 264 nm. The separation profiles in B were at optimal BGE. Sample: 1.0 mmol/L Leu, Ile and Val. Others as in Fig 1.

Indirect UV detection is an important approach in CE to detect weak or none UV absorption substances. The key is to find a suitable UV absorbing probe, *e*.*g*. stable in the electric field, high molar absorptivity, the similar mobility and the same charge with sample ions. In addition, we also studied the effects of β-CD (from 0 to 50 mmol/L) and pH (from 11.8 to 12.4) in order to achieve satisfactory results. We investigated six potential UV absorbing probes as p-aminobenzoic acid, 5-sulfosalicylic acid, 2,5-dihydroxy benzoic acid, 2-aminobenzimidazole, 4-aminobenzamide and PAS. Among these chemicals, PAS was finally considered as the proper UV absorbing probe that added in phosphate buffer system. As well known, the concentration of UV absorbing probe is an important factor to determine the LOD in indirect UV detection [25]. Fig 2A displayed the separation efficiency of BCAAs affected by the concentration of PAS varied from 5.0 to 20 mmol/L in 2.0 mmol/L Na_2_HPO_4_, 40.0 mmol/L β-CD at pH 12.2 (adjusted by 1.0 mol/L NaOH). The β-CD still acted as chiral selector. A lower concentration of PAS in BGE might contribute to a lower LOD, but the separation was significantly deteriorated when PAS at 5.0 mmol/L as Fig 2A. Therefore, 10 mmol/L PAS was used in the follow-up study. PAS exhibits two UV absorption peaks at 207 and 264 nm, where the molar absorptivities were calculated at 2.35×10^4^ and 1.27×10^4^ L•mol^-1^•cm^-1^, respectively. At the optimal BGE conditions, the indirect UV detection of BCAAs was carried on 207 and 264 nm as shown in Fig 2B. The sensitivity at 264 nm was higher than that at 207 nm. It was indicated that the detection sensitivity was not only depended on the molar absorptivity, but also related to the noise coefficient of the visualization agent [26]. Finally, the wavelength of 264 nm was selected in study.

At the optimal indirect UV detection, we found the resolution between Leu and L-Ile was around 1.0. The enantiomers of D- and L-Ile were not fully separated. The LODs, calibration range and precision thresholds were listed in Table 1. The averaged LOD was around 10.1 μmol/L, which is better than direct UV detection and the reported OPA derivatization method. The RSDs for migration time and peak area were below 0.9% and 3.7%. All above demonstrates that indirect UV detection was reliable and effective to analyze BCAAs.

### Quantification of BCAAs in sport nutritional supplements

**Fig 3:**
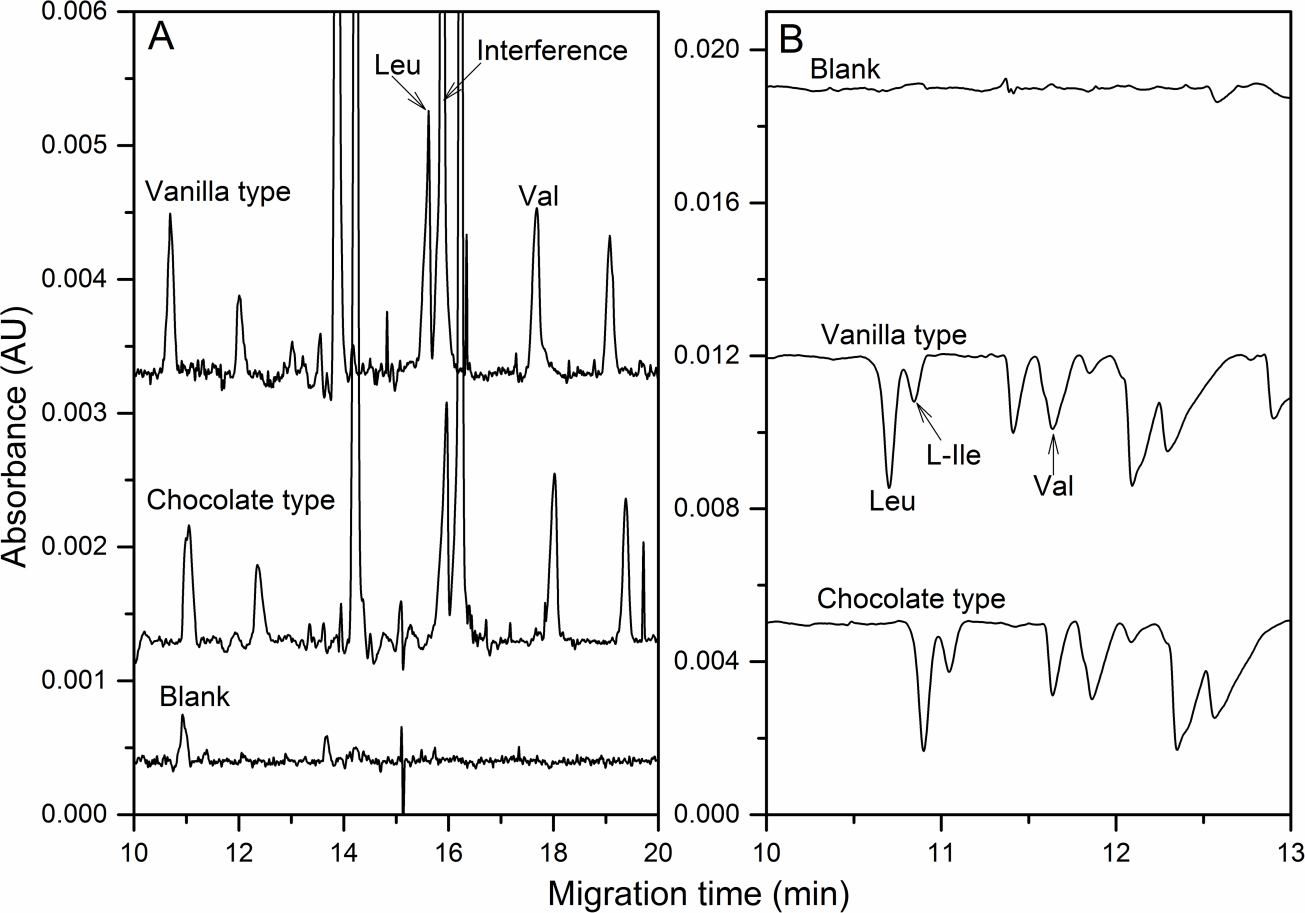
Identification of BCAAs in hydrolysates of the sports nutrition supplements by direct (A) and indirect (B) UV detection mode. The hydrolysates were 20- and 50-fold dilution, respectively for direct and indirect UV detection. Optimal BGE and others as in Figs 1 and 2.

To quantify the amount of BCAAs in sports nutritional supplements is important to direct supplementary consumption in sport science. As described in Section 2.3, two commercial sports nutritional supplements with different flavors were hydrolyzed for the quantitative analysis of BCAAs. The developed direct and indirect CE-UV detection methods were adopted to quantify the BCAAs in these two samples. Here, the peak identification in Fig 3 was based on the standard addition. The quantitative analysis of BCAAs was due to standard curve as listed in Table 1. Fig 3A exhibited the hydrolysates (20-fold diluted) of two supplemental proteins with direct UV detection. Leu and Val could be identified in the complex compositions, but some interference with high UV absorption overlapped with L-Ile. Therefore, only the amount of Leu and Val could be evaluated, *e*.*g*. around at 105 mg/g and 46 mg/g in vanilla type. Fig 3B presented the hydrolysates (50-fold diluted) that analyzed by indirect UV detection. The component of Leu, L-Ile and Val was identified by spiking of standards at different concentration. In this case, all three BCAAs could be quantified which listed in Table 2. Because this method could obtain good recoveries, it was proved to be effective and accurate. For example, the amounts of Leu and Val in Vanilla type obtained by direct and indirect UV detection were consistent. The tiny difference of migration time between vanilla and chocolate type might be caused by the additions in sample matrix for different flavors.

**Table 2:** Amounts of BCAAs in supplemental proteins with indirect UV detection.

Flavor type	Compositions	Amount (mg/g)	Recovery (%)
Vanilla	Leu	109.0±3.0	114.6
	L-Ile	36.5±2.1	92.3
	Val	48.7±2.1	103.3
Chocolate	Leu	113.8±4.0	116.1
	L-Ile	37.1±2.7	102.7
	Val	48.7±0.6	96.9

According to our results, the total amount of BCAAs in two supplemental proteins was fairly consistent. The total amount of BCAAs was at 194.2 and 199.6 mg/g in vanilla and chocolate type. In high quality whey protein, the content of BCAAs was reported around at 209 mg/g [27]. The obtained data seemed to match with the nutritional value since such two sports nutritional supplements were made from whey protein. World Health Organization (WHO) suggests 0.8 g whey protein per kg of body weight in one day (g/kg/d) would meet the BCAAs requirement of adult humans (in WHO Technical Report Series 935). For athletes, the needs of BCAAs are higher than those of sedentary persons. In general, the requirement of BCAAs is around 0.24~0.33 g/kg/d [28]. Otherwise, the renal function damage and loss of bone mass might happen if the BCAAs exceed to tolerable upper intake level. To determine the residual of BCAAs in urine would be useful to evaluate the consumption proper or not, the work is still ongoing.

## Concludings

In this work, the direct and indirect CE-UV detection methods were developed for the analysis of BCAAs. The targets could be directly detected at 195 nm without derivatization in an optimal BGE that contained with 40.0 mmol/L sodium tetraborate, and 40.0 mmol/L β-CD at pH 10.2. The β-CD was an effective chiral selector for the enantioseparation of Leu, D- and L-Ile. For the indirect UV detection at 264 nm, PAS was used as the UV absorbing probe. The used BGE was 2.0 mmol/L Na_2_HPO_4_, 10.0 mmol/LPAS, and 40.0 mmol/L β-CD at pH 12.2. The repeatability (n = 6) of migration time and peak area were acceptable for qualitative and quantitative analysis of BCCAs. The obtained LODs (tens μmol/L) of this developed method were not noticeable, but were comparable with those of inline derivatization methods with the same UV detection. Without previous tedious sample cleaning-up step as solid-phase extraction and derivatization process (from several to thirty minutes), the analysis was achieved within 20 minutes in a simple and rapid process. The applicability of two proposals was validated in the analysis of BCAAs in the supplemental proteins. The total amount of BCAAs was quantified at 194.2 and 199.6 mg/g, respectively in vanilla and chocolate type. The results are consistent with the amount of BCAAs in high quality whey protein. The developed methods are feasible in determination of BCAAs that presents in supplemental proteins or bioliquids.
